# Recent insights into the use of immune checkpoint inhibitors in gastric cancer

**DOI:** 10.1097/j.pbj.0000000000000162

**Published:** 2022-02-08

**Authors:** Soraia Rodrigues, Ceu Figueiredo

**Affiliations:** a Department of Pathology, Pathology and Oncology Unit, Faculty of Medicine of the University of Porto; b Instituto de Investigação e Inovação em Saude, Universidade do Porto (i3S); c Instituto de Patologia e Imunologia Molecular da Universidade do Porto (Ipatimup), Porto, Portugal.

**Keywords:** biomarkers for immunotherapy, gastric cancer, immune checkpoint inhibitors, molecular classification

## Abstract

Gastric cancer (GC) is the fifth most incident and the fourth deadliest cancer worldwide. GC is a heterogeneous disease from the histological and molecular standpoints. This malignancy is mostly diagnosed at advanced stages of the disease, where the available therapeutic interventions are not effective. The emergence of immunotherapy has transformed the landscape of cancer treatment, including GC, and currently immune checkpoint inhibitors have been approved for the treatment of patients with recurrent/metastatic GC. This review summarizes the main clinical trials evaluating the use of immune checkpoint inhibitors in GC. It also highlights the potential of biomarkers for patient selection for GC immune checkpoint inhibition therapy, including programmed cell death ligand 1 expression and tumor mutational burden, and characteristics of the GC molecular classification, such as microsatellite instability status and Epstein-Barr virus infection, as predictors of response to blockade of the programmed cell death 1/programmed cell death ligand 1 axis.

## Introduction

Gastric cancer (GC) is among the cancers with the most impact in all societies. It is the fifth most incident malignant disease worldwide, with a geographically heterogeneous incidence. The highest rates are observed mainly in Eastern Asia (especially in Korea, Mongolia, Japan, and China), Europe (central and eastern), and South America, and the lowest incidence rates in Africa and Northern America.^1,2^ GC is also the fourth main cause of death by cancer in the world.^1^ The great majority of cases experience a late diagnosis, mostly due to the lack of solid and global screening strategies and to the lack of specific symptoms.^3^ Consequently, a significant proportion of patients present with advanced stage tumors.^4^ The prognosis for advanced stages of this disease continues to be dire, and the 5-year survival is 25% to 30%.^5–7^

There are various approaches to GC therapy, including perioperative, adjuvant, and palliative chemotherapy, and tumor endoscopic/surgical resection, none of which is fully effective.^4^ Targeted therapy has been also introduced for a particular subset of GCs that overexpress human epidermal growth factor receptor 2 (HER2).^7–9^ More recently, a great deal of attention is being given to immunotherapy, which may be used in various types of cancers, including GC.^10^

## Gastric cancer heterogeneity

GC comprises various types of tumors. The great majority arise in glandular structures and are classified as adenocarcinomas.^11^ In addition, mesenchymal tumors and B-cell lymphomas can also be found, although to a much lesser extent.^12,13^ Herein GC will be used as synonym of gastric adenocarcinoma.

GC is a heterogeneous disease from both histological and molecular standpoints. Histologically, and according to the Laurén's classification, there are 2 main GC subtypes.^14^ The intestinal subtype is usually diagnosed in older patients, most frequently in men, and appears in the distal part of the stomach, with a frequently exophytic growth pattern. The main histological characteristic of these tumors is the formation of glands and the synthesis of extracellular mucins. The diffuse subtype affects younger patients of both sexes equally, and arises mainly, although not exclusively, in the gastric body, frequently with *linitis plastica* growth pattern. Histologically, diffuse GC is characterized by the loss of cellular cohesion and the presence of isolated cells that contain high quantities of intracytoplasmic mucins (“signet ring cells”).^14^ An additional and rarer GC subtype, comprises tumors that present characteristics of both the intestinal and diffuse subtypes, and is denominated mixed type GC. According to the World Health Organization, GC comprises 5 main histological subtypes: papillary, tubular, poorly cohesive (characterized by “signet ring cells”), mucinous (when mucinous pools exceeds 50% of the tumor), and mixed adenocarcinomas. Other less common variations exist, such as the squamous cell, adenosquamous, hepatoid, and medullary carcinomas.^11,15^ Despite the heterogeneity referred above, histological subtypes have not provided significant contribution to therapeutic decisions.^16^

From the molecular standpoint, GC is also heterogeneous. The so-called “Singapore-Duke” classification considers 3 GC subtypes.^17^ The proliferative GC subtype that corresponds to the intestinal type, presenting high *TP53* mutations, high copy number alterations, and oncogenic activation. The mesenchymal GC subtype that corresponds to the diffuse type, having low copy number alterations, low number of *TP53* mutations, and a high activity of the epithelial-mesenchymal transition (EMT) pathway, similarly to stem cells. Finally, the metabolic GC subtype is characterized by low number of *TP53* mutations, high activity of the spasmolytic polypeptide-expressing metaplasia pathway, and high activity of metabolic pathways.^17,18^

The Cancer Genome Atlas (TCGA) classification considers 4 GC subtypes.^19^ The Epstein-Barr virus (EBV)-infected (EBV+) subtype constitutes 9% of all GCs and is more frequent in men and in younger patients. These tumors appear mainly in the upper part of the stomach, specifically, in the *gastric fundus.* Histologically, EBV+ GC is moderate to poorly differentiated, usually with dense lymphocytic infiltration.^20^ Molecular characteristics of EBV+ GC include extreme hypermethylation, *CDKN2A* methylation, but without *MLH1* methylation, and *PIK3CA* and *ARID1A* mutations. The EBV+ GC subtype is also characterized by amplification of *JAK2, ERBB2,* and programmed cell death ligand ½ (PD-L1/2), the latter with an important role as targets of immunotherapy in the treatment of GC. An additional characteristic of this tumor subtype is the enhancement in immune cell signaling pathways.^21^

Microsatellite unstable (MSI) tumors make up to 15% to 30% of all GCs, and are more frequent in women and older patients. They arise mainly in the lower part of the stomach, particularly in the gastric antrum. The histology of MSI GC is similar to that of the intestinal subtype. MSI tumors are diploid and hypermutated, with mutations in *ARID1A, PIK3CA, PTEN, ERBB2, ERBB3, EGFFR, KRAS, RNF43,* and *MHC1. TP53* mutations are frequent and *MLH1* is hypermethylated. MSI tumors are enriched in mitotic and DNA damage pathways.^21^

Genomically stable (GS) tumors constitute 20% of all GCs. GS tumors affect both sexes equally and have an early onset diagnosis compared to other subtypes of GC. They are mainly located in the antrum and comprise histologically diffuse tumors. GS tumors are characterized by mutations in *CDH1, ARID1A,* and *RHOA,* by *CLDN18-ARHGAP* fusion, and by high cell adhesion and angiogenesis pathways expression. In this subtype, *TP53* mutations are not common.^21^

Chromosomally unstable (CIN) GC comprises 50% of the cases.^20^ CIN tumors originate in the gastric fundus and esophagogastric junction. Histologically, CIN tumors can be of the intestinal subtype, when associated with gains in copy number of 8q, 17q, and 20q, and of the diffuse subtype, when associated with gains of 12q and 13q.^20^ CIN tumors are aneuploid and harbor genomic amplifications in: *RTKs* and *KRAS,* which are mutually exclusive; transcription factors, including *KLF5, GATA4, GATA6,* and *OCT1;* cell cycle mediators, including *CCNE1, CCND1,* and *CDK6.* Mutations in *HER2, BRAF, EGFR, MET, FGFR2,* and *RAS* have also been identified. Unlike the other subtypes, CIN GC shows a high frequency of *TP53* mutations.^21^

The Asian Cancer Research Group classification also considers 4 GC subtypes, which unlike the TCGA classification, may predict disease progression or disease prognostic.^18,21–23^ EMT/ microsatellite stable (EMT/MSS) comprises 15% of all GCs, it is located in the gastric body, and is histologically similar to the diffuse subtype. EMT/MSS presents EMT gene expression signature and loss of *CDH1* expression. This subtype corresponds to the worst prognostic. The MSI subtype represents 23% of tumors, present in the antrum, and is closer to histological intestinal subtype. An MSI gene expression signature, together with loss of *MLH1* expression and hypermutation is observed in this GC subtype that is associated to a better prognosis. The MSS/ TP53+ and the MSS/TP53—subtypes constitute respectively 26% and 36% of GCs. These subtypes are distinguished by the activation of *TP53,* in which MSS/TP53+ has an intact *TP53* gene. Overall, the characteristics associated to specific GC subtypes can potentially provide novel therapeutic targets as well as new means for patient stratification.^21^

## Gastric cancer immunotherapy using immune checkpoint inhibitors

Immunotherapy has revolutionized cancer treatment. Among the different forms of immunotherapy, immune checkpoint inhibitors are the best studied and the most used therapeutic agents.^24^ Malignant tumors frequently use mechanisms of immune suppression and tolerance to prevent immune destruction. The idea underlying immune checkpoint blockade therapy is the removal of signals that inhibit T-cell activation and effector functions, which in turn allows the establishment of an efficient anti-tumor response.^24^

The pioneer immune checkpoint inhibitor was ipilimumab, an anti-cytotoxic T-lymphocyte–associated antigen-4 monoclonal antibody, originally approved for the treatment of unresectable/ metastatic melanoma.^25^ Following this breakthrough, other molecules for immune checkpoint inhibition followed, including nivolumab, pembrolizumab, and avelumab, which target the programmed cell death 1 (PD-1)/PD-L1 axis.^26–28^ In the context of GC, some of these immune checkpoint inhibitors were approved as a third-line therapy in advanced or recurrent GC, whereas their use as first-/second-line options has been and still is under evaluation. Results of major clinical trials are summarized in Table 1.^26–33^

**Table 1:** Summary of results of selected clinical trials that evaluate the use of immune checkpoint inhibitors in gastric cancer

Trial namereference	Phase/line	Agent	Target	PD-L1	Treatment arms	Number patients	Primary endpoint	OS (mo)	PFS (mo)	ORR (%)
ATTRACTION-2^27^	III/ ≥3L	Nivolumab	PD-1	Unselected	Nivolumab	330	OS	5.26	1.6	11.2
					Placebo	163		4.14	1.5	0
KEYNOTE-59^26^	II/ ≥3L	Pembrolizumab	PD-1	Unselected	Pembrolizumab	259	ORR	5.6	2	11.6
JAVELIN Gastric 300^28^	III/ 3L	Avelumab	PD-L1	Unselected	Avelumab	185	OS	4.6	1.4	2.2
					CT^1^	186		5.0	2.7	4.3
CHECKMATE-032^31^	I-II/ ≥3L	Nivolumab	PD-1	Unselected	Nivolumab	59	ORR	6.2	1.4	12
					Nivolumab 1 + ipilimumab 3	49		6.9	1.4	24
					Nivolumab3 + ipilimumab1	52		4.8	1.6	8
KEYNOTE-61^29^	III/ 2L	Pembrolizumab	PD-1	Positive	Pembrolizumab	196	OS, PFS	9.1	1.5	16
					Paclitaxel	199		8.3	4.1	14
KEYNOTE-62^30^	III/ 1L	Pembrolizumab	PD-1	Positive	Pembrolizumab	256	OS, PFS	10.6 CPS ≥1; 17.4 CPS ≥10	2.0	14.8
					Pembrolizumab + CT^2^	257		12.5 CPS ≥1; 12.3 CPS≥10	6.9	48.6
					CT^2^	250		11.1 CPS≥1; 10.8 CPS≥10	6.4	37.2
CHECKMATE-649^32^	lII/ 1L	Nivolumab	PD-1	Positive	Nivolumab + CT^3^	473	OS, PFS in CPS ≥5	14.4	7.7	-
					CT^3^	482		11.1	6.1	-
ATTRACTION-4^33^	II-III/ 1L	Nivolumab	PD-1	Unselected	Nivolumab + CT^4^	362	PFS, OS	17.5	10.5	57.5
					Placebo + CT^4^	362		17.2	8.3	47.8

1L, first line; 2L, second line; 3L, third line; ≥3L, third line or later; CPS, combined positive score; CT, chemotherapy; CT^1^ included paclitaxel or irinotecan; CT^2^ included cisplatin plus fluorouracil or capecitabine; CT^3^ included capecitabine plus oxaliplatin or fluorouracil, leucovorin, and oxaliplatin; CT^4^ included S-1 plus oxaliplatin or capecitabine plus oxaliplatin; NR, not reached; ORR, objective response rate; OS, overall survival; PD-1, programmed cell death 1; PFS, progression-free survival.

Nivolumab is a human monoclonal IgG4 kappa antibody that binds to the PD-1 receptor and blocks its interaction with PD-L1 and PD-L2. Nivolumab was initially approved in Japan for the treatment of several different types of cancers.^34^ The randomized, double-blind, placebo-controlled, phase III ATTRACTION-2 trial assessed the efficacy and safety of nivolumab in patients with unresectable advanced/recurrent gastric or gastro-esophageal junction (GEJ) cancer, who had been treated with 2 or more chemotherapeutic regimens.^27^ The trial enrolled 493 Asian patients from Japan, South Korea, and Taiwan, who were randomly assigned to receive nivolumab or placebo. The results showed that nivolumab improved the overall survival (OS) in patients with GC refractory to standard chemotherapy; the median OS was 5.26 months in the nivolumab group, contrasting with 4.14 months in the placebo group. In this study, the 1-year OS rate was 26.2% in patients receiving nivolumab, in comparison with 10.9% in patients receiving placebo. The updates of the trial showed that the 2- and the 3-year OS rates were, respectively, 10.6% and 5.6% for nivolumab, and 3.2% and 1.9% for placebo, and confirmed the long-term efficacy of nivolumab.^35,36^ Following the first results of the ATTRACTION-2 trial, nivolumab was also approved in various Asian countries as a third-line or later option in patients with unresectable advanced or recurrent gastric or GEJ cancer.^35^ One of the limitations of the trial was that the patient population consisted only of Asian patients.^37,38^

The KEYNOTE-59, open-label, phase II trial, evaluated the safety and efficacy of monotherapy with pembrolizumab, a humanized monoclonal IgG4 kappa antibody that binds to the PD-1 receptor, blocking its interaction with PD-L1 and PD-L2.^26^ The trial involved a cohort of 259 patients from 16 international locations with previously treated advanced GC or GEJ cancer.^26^ The objective response rate (ORR) associated with pembrolizumab treatment was 11.6%, 2.3% of the patients had complete response, and the median OS was 5.6 months. These results favored further developments of the use of pembrolizumab monotherapy in patients with advanced gastric or GEJ cancer with ≥2 previous lines of treatment. Based on the outcomes of this trial, the Food and Drug Administration (FDA)-approved pembrolizumab for treatment of recurrent and locally advanced or metastatic GC.^39^

An additional immune checkpoint inhibitor is avelumab, a human IgG1 lambda monoclonal antibody that by binding PD-L1, blocks its interactions with PD-1 and B7.1 receptors. The use of avelumab as third-line therapy was investigated in the JAVELIN Gastric 300 phase III trial, which enrolled 371 patients with recurrent locally advanced or metastatic gastric or GEJ cancer that were randomized to receive avelumab or the physician's choice of third-line chemotherapy.^28^ Although the safety profile of avelumab was better than that of chemotherapy, the trial was not successful in meeting the primary or secondary end points; the median OS was 4.6 in the avelumab group and 5 months in the chemotherapy group; the progression-free survival (PFS) was 1.4 versus 2.7 months and the ORR was 2.2% versus 4.3% in the avelumab versus chemotherapy arms, respectively. Therefore, in the third-line setting, the use of avelumab as a single agent did not improve OS or PFS in comparison chemotherapy.

The CheckMate-032 phase I/II trial tested the efficacy of nivolumab and ipilimumab in patients with chemotherapy-refractory metastatic gastric or GEJ cancer, in 160 patients from centers in the United States and Europe.^31^ Patients were treated with nivolumab, or with combinations of nivolumab plus ipilimumab. The ORR, median PFS, and OS were, respectively, 12%, 1.4 months, and 6.2 months in the patients receiving nivolumab, 24%, 1.4 months, and 6.9 months in those receiving nivolumab 1 mg/kg and ipilimumab 3 mg/kg, and 8%, 1.6 months, and 4.8 months with the combination of nivolumab 3 mg/kg and ipilimumab 1 mg/kg. Although the combination of nivolumab 1 mg/kg plus ipilimumab 3 mg/kg had a numerically higher ORR than that of patients treated with nivolumab monotherapy, the median OS was similar in these patient groups. Nevertheless, the study demonstrated a reasonable safety profile and long-lasting responses. In addition, the clinical benefits of nivolumab monotherapy were similar to those of the ATTRACTIONS-2, suggesting consistent therapeutic benefit across patients from Asian and Western countries.

Although now established as a third-line therapy, immune checkpoint inhibitors have not been so successful in earlier lines of therapy for GC. The open-label, phase III KEYNOTE-061 trial enrolled 592 patients with advanced gastric or GEJ cancer that progressed on first-line chemotherapy, with a PD-L1 combined positive score (CPS) ≥1.^29^ Patients were randomized to receive pembrolizumab or paclitaxel. Pembrolizumab did not significantly improve the OS compared with paclitaxel (9.1 vs 8.3 months) or PFS (1.5 vs 4.1 months), although it had a better safety profile.^29^

The following randomized, controlled, phase III KEYNOTE-062 trial enrolled 763 patients with untreated, locally advanced, unresectable, or metastatic gastric or GEJ cancer, with a PD-L1 CPS ≥1.^30^ Patients were randomized to receive pembrolizumab, pembrolizumab plus chemotherapy, or chemotherapy plus placebo. The OS of patients with PD-L1 CPS ≥1 treated with pembrolizumab was noninferior to that of patients treated with chemotherapy (10.6 vs 11.1 months). Interestingly, in patients with PD-L1 CPS ≥10 pembrolizumab extended OS versus chemotherapy (17.4 vs 10.8), but without statistical significance. Pembrolizumab plus chemotherapy was not superior to chemotherapy for OS in patients with CPS ≥1 (12.5 vs 11.1 months) or with CPS ≥10 (12.3 vs 10.8 months), or for PFS in patients with CPS ≥1 (6.9 vs 6.4 months).^30^

The ongoing randomized, phase II/III ATTRACTION-4 evaluates nivolumab plus chemotherapy versus placebo plus chemotherapy as a first-line treatment in HER2-negative, advanced, or recurrent gastric or GEJ cancer in Asian patients.^33^ The results of the double-blind phase III part demonstrated a statistically significant improvement in PFS in patients receiving nivolumab plus chemotherapy in comparison with the other study arm (10.5 vs 8.3 months), reaching one of the primary endpoints of the trial. However, no differences in OS, which was the other primary study endpoint, were observed between the 2 groups (17.5 vs 17.2 months). Similarly, the randomized, open-label, phase III CheckMate-649 trial compared nivolumab plus chemotherapy with chemotherapy alone as a first-line treatment for patients with advanced or metastatic gastric or GEJ cancer. The first results of the trial that enrolled 1581 patients from geographic locations worldwide demonstrated that in patients with tumors with PD-L1 CPS ≥1 there was statistically significant benefit in OS those treated with nivolumab plus chemotherapy versus those treated with chemotherapy alone (14.0 vs 11.3 months). In particular, in patients with tumors expressing PD-L1 with CPS ≥5, nivolumab plus chemotherapy showed statistically significant improvements in both OS and PFS in comparison with chemotherapy alone.^32^ Based on the results of the CHECKMATE-649 trial, the European Medicines Agency validated^40^ and the US FDA accepted for priority review^41^ the application of nivolumab combined with chemotherapy as first-line treatment in metastatic GC, GEJ cancer, and esophageal adenocarcinoma.

## Biomarkers for immune checkpoint inhibition in gastric cancer

The use of biomarkers for patient selection for immune checkpoint inhibition therapy aims to increase its efficacy, while reducing useless therapeutic exposure and health-related costs. In this section, we will summarize knowledge on biomarkers that are presently being tested in clinical trials addressing immune checkpoint blockade in GC.

### PD-L1 expression

PD-L1 expression is the most widely studied biomarker for patient selection for PD-1/PD-L1 therapy. The usefulness of PD-L1 expression as a biomarker has been reported in various large clinical trials that assessed PD-1 and/or PD-L1 inhibitors in melanoma, non–small cell lung cancer, and urothelial carcino-ma.^42–44^ In these studies, higher expression levels of PD-L1, as evaluated by immunohistochemistry, were predictive of response to therapy with PD-1 and/or PD-L1 inhibitors. However, for other cancer types, including renal cell carcinoma and hepato-cellular carcinoma, PD-L1 expression did not show to be a good biomarker.^45,46^

In GC, between 25% and 65% of tumors express PD-L1, and multiple mechanisms have been associated with PD-L1 upregu-lation, including *PDL1* gene amplification, structural variations in the 3’UTR of *PDL1*, polymorphisms in *PDL1* promoter, activation of oncogenic PI3K signaling, and cytokine- and chemokine-mediated regulation.^47,48^ PD-L1 expression in GC has been associated with high density of tumor-infiltrating lymphocytes, with MSI, and with EBV infection.^49,50^

The relationship between PD-L1 expression and prognosis in GC is controversial, and although some studies reported increased PD-L1 expression associated with adverse prognosis, others have shown a relationship with better patient outcome, or report that PD-L1 expression is not a prognostic factor.^49–51^ Several meta-analyses have been now been performed to examine the clinicopathological and prognostic significance of PD-L1 expression. A meta-analysis that included 10 studies and 1901 patients with GC from Asia indicated that PD-L1 expression was associated with a shorter OS.^47^ The expression of PD-L1 was also associated with tumor size, and lymph node metastasis, but not with age or sex, tumor differentiation, invasion depth, or tumor stage. A more recent meta-analysis including 15 studies and 3218 patients from China, South Korea, Japan, and Germany, showed that PD-L1 expression was associated with a decrease in the 3-and 5-year survival rates.^52^ In the subgroup analyses of ethnicity, PD-L1 expression in Asian patients was also associated with a decrease in the 3- and 5-year survival rates. PD-L1 expression was associated with lymph node metastasis but not with tumor staging. These results point to the possibility of using PD-L1 expression as GC biomarker for PD-1/PD-L1-targeted therapy.

It is important, however, to mention that major problems exist regarding the comparisons between studies, namely the use of different antibodies, assays, or devices for PD-L1 immunohis-tochemistry, as well as differences in scoring criteria.

In the 3-year update of the ATTRACTION-2 trial, PD-L1 was retrospectively analyzed using the PD-L1 IHC 28-8 pharmDx assay. In the 192 patients who had available tumor tissue, no differences were found regarding the efficacy of nivolumab compared to that of placebo in patients’ OS.^27,36^ In CHECKMATE-032, the benefits of nivolumab or the combinations of nivolumab with ipilimumab were observed, no matter the immunohistochemical status of PD-L1 independently.^31^ Finally, in the JAVELIN Gastric 300 that used the PD-L1 IHC 73-10 pharmDx assay, no differences were identified in the outcomes of avelumab treatment between patients with PD-L1-positive or -negative tumors. In these trials, however, the scoring of PD-L1 was performed using a tumor proportion score of ≥1%, which considers expression of PD-L1 in 1% or more of tumor cells.^53^

The trials that assessed pembrolizumab in GC showed efficacy of this immune checkpoint inhibitor in PD-L1-positive tumors, using the FDA-approved PD-L1 IHC 22C3 pharmDx assay, which scores PD-L1 expression in tumor cells, lymphocytes, and macrophages (CPS).^26,29,30,53^ In KEYNOTE-59, the ORR and the median response duration of patients with PD-L1-positive tumors were 22.7% and 8.1months, whereas the responses were significantly lower, 8.6% and 6.9months, respectively, in patients with tumors that were PD-L1 negative.^26^ In KEYNOTE-61, the ORR for patients treated with pembrolizumab versus paclitaxel was 16% versus 14% in patients with CPS ≥1 tumors, but in subgroup analysis, ORR was 25% versus 9% in the PD-L1 CPS ≥10 subgroup, and 2% versus 10% in the PD-L1 CPS <1 subgroup.^29^ In KEYNOTE-062, the OS for patients treated with pembrolizumab was 10.6 months in those with PD-L1 CPS ≥1 tumors, and a prolonged, though not statistically tested, OS of 17.4 months was observed in patients with PD-L1 CPS ≥10 tumors.^30^

Very recently, and to better define CPS specificity as a predictor of clinical outcome, Wainberg et al^54^ comprehensively studied in post-hoc analyses the efficacy of pembrolizumab in patients with CPS ≥10 in the 3 trials mentioned above. In KEYNOTE-059 (median follow-up 6 months), median OS was 8 months, ORR was 17%, and median duration of response (DOR) was 21 months. In KEYNOTE-061 (median follow-up 9 months), median OS (pembrolizumab vs chemotherapy) was 10 versus 8 months, median PFS was 3 versus 3 months, ORR was 25% versus 9%, and median DOR was not reached versus 7 months. In KEYNOTE-062 (median follow-up 11 months), median OS (pembrolizumab vs chemotherapy) was 17 versus 11 months, median PFS was 3 versus 6 months, ORR was 25% versus 38%, and median DOR was 19 versus 7 months. This shows that more favorable clinical outcomes are consistently observed in first, second, and third lines of pembrolizumab therapy in patients with PD-L1 CPS ≥10 tumors, and suggest that PD-L1 expression could be used to identify patients who would benefit from PD-1/ PD-L1-targeted therapy.

### Microsatellite instability

The accumulation of mutations in microsatellite regions of the genome, which are repeated sequences of nucleotides where DNA polymerase is more prone to replication errors, is known as microsatellite instability (MSI). MSI is generally caused by a deficiency in mismatch repair systems.^55,56^

About 20% to 25% of gastric tumors have the MSI phenotype.^19,57,58^ Patients with MSI GC have better prognosis than those with MSS tumors.^57,59,60^ MSI tumors have distinct clinicopathologic features and are associated with older age, female sex, and Laurén's intestinal histology.^57,58^ A meta-analysis that included 48 studies and 18.612 patients, of which 9.2% had MSI tumors, confirmed the relationship between MSI tumors and these features, but also with the absence of lymph node metastases, and stages I–II (TNM classification). Patients with MSI tumors also had better OS than patients with MSS GC.^60^

MSI and mismatch repair deficiency in tumor cells may lead to higher levels of mutations and the appearance of immunogenic neoantigens, leading to easier recognition by immune cells. This may facilitate the action of immune checkpoint inhibitors, as these types of tumors exhibit a high density of immune cells. Accordingly, in comparison with MSS GC, MSI gastric tumors have higher numbers of PD-L1-positive tumor and immune cells, and increased number of tumor-infiltrating lymphocytes.^50,61,62^

In KEYNOTE-59, 67% of the enrolled patients were assessed for GC MSI, of which 4% had MSI-high tumors. In patients with MSI-high GC, the ORR to pembrolizumab treatment was 57.1%, contrasting with an ORR of 9% for patients with non– MSI-high GC.^26^ In CHECKMATE-32, and in all study arms, there were significantly better responses in patients with MSI-high GC compared to non–MSI-high patients.^31^ The ORRs for MSI-high versus non–MSI-high were for nivolumab: 29% versus 11%; for nivolumab 1 mg/kg plus ipilimumab 3 mg/kg: 50% versus 19%; and for nivolumab 3 mg/kg plus ipilimumab 1 mg/kg: 50% versus 5%. The OS for MSI-high versus non–MSI-high were for nivolumab: 57% versus 33%; for nivolumab 1 mg/kg plus ipilimumab 3 mg/kg: 50% versus 32%; and for nivolumab 3 mg/kg plus ipilimumab 1 mg/kg: 50% versus 23%.

In post-hoc analysis of the patients enrolled in KEYNOTE-61, those with MSI tumors had superior responses to pembrolizumab (47%, regardless of the PD-L1 CPS), compared to 17% in the paclitaxel group.^29^ In CHECKMATE 62, OS was enhanced in patients with MSI tumors with PD-L1 CPS ≥1, and overall outcomes proved more efficient in the MSI-high population. The predictive value of PD-L1 CPS ≥10 remained constant, regardless of the MSI status, which demonstrates the independent value of both biomarkers.

A recent meta-analysis of randomized clinical trials evaluated the role of MSI as a positive predictive factor for PD-1 immunotherapy as first- or second-line regimens in patients with advanced GC.^63^ The study included data from KEYNOTE-061, CHECKMATE-649, JAVELIN Gastric 100, and KEYNOTE-062,^29,30,32,64^ and provided evidence of improved survival and response in advanced patients with GC with MSI-high tumors who received anti-PD-1 blockade, with significantly greater OS compared with patients with MSS tumors.

Also recently, a post-hoc analysis of 1614 patients, 84 of which had MSI-high gastric or GEJ cancer, and enrolled in the KEYNOTE-059, KEYNOTE-061, and KEYNOTE-062 trials, assessed the antitumor effects of pembrolizumab versus chemotherapy, irrespectively of the line of therapy.^65^ Results from this study showed that pembrolizumab alone, or combined with chemotherapy, was associated with prolonged OS and PFS and with durable responses in comparison to chemotherapy alone, suggesting the MSI-high status as biomarker for patient selection, irrespectively of the line of therapy in which it is received.

### EBV infection

As discussed above, the TCGA identified EBV-positive tumors as a distinct GC subgroup.^19^ Among other features, these tumors are characterized by rich lymphocytic infiltrates, containing CD8-positive cytotoxic T cells and high number of mature dendritic cells, and are enriched in immune cell signaling pathways.^49,66^ Furthermore, about 15% of EBV-positive GCs have amplification of the PD-L1- and PD-L2-encoding genes, and have PD-L1 expression in both tumor cells and immune cells.^19,50,67^ These features suggest that EBV-positive GC may be more susceptible to PD-1/PD-L1 blockade.

A case report described response to avelumab treatment in 1 EBV-positive GC patient.^68^ In a clinical trial that evaluated the impact of toripalimab (an anti-PD-1 antibody) on 55 advanced GC patients, of the 4 EBV-positive patients, 1 case had partial response, 2 cases had stable disease, and 1 case had progressive disease.^22^ In a prospective phase 2 clinical trial of 61 patients with metastatic GC that had been treated with pembrolizumab, whereas the general ORR was 24.6%, in the 6 patients with EBV-positive tumors the ORR was 100%, all responding to pembrolizumab.^69^ In this trial, there were also 7 patients with MSI-high GC and, in this group, the ORR was 85.7%. These finding suggest that EBV-positive GC patients may derive benefit from pembrolizumab therapy. Large prospective clinical trials are needed to evaluate EBV positivity as a biomarker for GC immune checkpoint therapy.

### Tumor mutational burden

During cancer initiation and progression, tumor cells acquire thousands of different mutations. Nonsynonymous mutations will cause tumors to express neoantigens, which are tumor cell specific and will distinguish them from normal cells.^70^ Epitopes of these mutant proteins can be expressed at the cancer cell surface, thus rendering these cells recognizable as foreign by T cells. It has been shown that the tumor mutational burden (TMB), and consequently the neoantigen formation potential in a certain tumor, will determine the effectiveness of the response to immunotherapy, as highly mutated cells can be distinguished and, therefore, targeted more proficiently.^70,71^ Accordingly, melanoma and non–small cell lung cancer, the 2 tumor types with highest prevalence of somatic mutations,^72^ have excellent responses to immune checkpoint blockade.^73–76^ Interestingly, in a study that evaluated almost 9900 samples of 35 cancer types, no significant correlations between TMB and PD-L1 expression within most cancer subtypes were observed, suggesting that each may be used as a biomarker for predicting the response to immune checkpoint blockade.^77^

The relationship between TMB and response to therapy with pembrolizumab has been analyzed in a study that involved multiple cohorts of patients with different types of solid tumors.^78^ Objective responses were observed in 29% of patients with TMB-high status (≥10 mutations per megabase), in comparison to only 6% in patients with non–TMB-high. Noteworthy, TMB had predictive value, regardless of the tumor PD-L1 expression and of the MSI status.

In a study of metastatic gastrointestinal cancer patients from China, including 57 patients with GC, treated with immune checkpoint inhibitors, patients with higher TMB had longer OS than those with lower TMB.^79^ In another study of 63 South Korean patients with advanced GC treated with pembrolizumab or nivolumab, responders had significantly higher TMB than nonresponders with stable disease.^38^ In survival analysis, patients with high TMB had longer PFS. Although in univariate analysis, TMB, MSI, response to treatment, and ECOG performance status were all significantly associated with PFS, in multivariate analysis, both TMB-high and the ECOG ≤1 remained independent predictors of longer PFS.

In a clinical trial that analyzed toripalimab therapy (a PD-1 antibody) in advanced GC, patients with TMB-high had significant higher OS (14.6 months) than those with TMB-low (4.0 months) with patients.^80^ Patients with TMB-high versus TMB-low also had enhanced ORR (33.3% vs 7.1%), and a numerically longer PFS, but without statistical significance. The analysis of the TMB in patients of KEYNOTE-061 showed that TMB ≥10 mut/Mb had a positive association with ORR, PFS, and OS in patients treated with pembrolizumab but not with paclitaxel.^81^ Interestingly, the OS benefits of pembrolizumab versus paclitaxel in TMB ≥10 mut/Mb remained, when patients with MSI-high tumors were excluded. Taken together, and although needing more consolidated data, the TMB appears to be a promising biomarker for GC immunotherapy.

## Conclusions

The outcome of GC, in particular of advanced disease stages, remains poor. Immunotherapy based on immune checkpoint inhibition in advanced GC has shown promising benefits, in particular when patients who will derive most benefit from this type of therapy are selected. The heterogeneity of GC and the identification of GC subtypes with distinct molecular profiles has offered the opportunity to discover not only new GC therapeutic targets but also novel markers of response to immune checkpoint blockade. Nevertheless, further research is needed, as a lot of uncertainty still exists regarding immune checkpoint inhibitors and biomarker effectiveness. Future approaches should also consider additional biomarkers to identify patients who could better respond to the different inhibitors, thus contributing to improve the negative prognosis associated with advanced GC.

## Acknowledgments

Financial support and sponsorship: none.

## Author contributions

Conception and design: CF; Acquisition of data and interpretation: SR and CF; Drafting of manuscript: SR and CF; Revising of manuscript for important intellectual content: CF.

## Conflicts of interest

The authors declare no conflicts of interest.
